# Trends in parasite prevalence following 13 years of malaria interventions on Bioko island, Equatorial Guinea: 2004–2016

**DOI:** 10.1186/s12936-018-2213-9

**Published:** 2018-02-05

**Authors:** Jackie Cook, Dianna Hergott, Wonder Phiri, Matilde Riloha Rivas, John Bradley, Luis Segura, Guillermo Garcia, Chris Schwabe, Immo Kleinschmidt

**Affiliations:** 1 MRC Tropical Epidemiology Group, London School of Hygiene and Tropical Medicine, London, WC1E7HT UK; 2 Medical Care Development International, Silver Spring, MD USA; 3 Bioko Island Malaria Control Project, Medical Care Development International, Malabo, Equatorial Guinea; 4 Equatorial Guinea Ministry of Health and Social Welfare, Malabo, Equatorial Guinea; 5 School of Pathology, Faculty of Health Sciences, University of Witwatersrand, Johannesburg, South Africa

**Keywords:** Malaria, Vector control, Longitudinal, Epidemiology, Intervention, Monitoring and evaluation

## Abstract

**Background:**

Whilst there have been substantial reductions in malaria transmission over the past decade, in many countries in West and Central Africa the malaria burden remains high. Monitoring and evaluation of malaria transmission trends and intervention strategies are key elements for malaria control programmes. This study uses a time series of annual malaria indicator surveys to track the progress of malaria control in Bioko Island, Equatorial Guinea, over a 13 year period of intensive interventions. Malaria infection and haemoglobin were measured annually in children (1 to 14 years) in cross-sectional household surveys from 2004 to 2016 in 18 sentinel sites across the island. Trends in transmission patterns were assessed and the impact of the vector control interventions (net use and spray coverage) was evaluated.

**Results:**

Between 2004 and 2016 approximately 106,500 individual tests for parasitaemia were conducted using rapid diagnostic tests. Although spray coverage remained relatively high (> 70%) over the time period, reported net usage was generally below 40%. Parasite prevalence reduced from 43.3 to 10.5% between 2004 and 2016. The prevalence of moderate to severe anaemia in children aged 1–5 years reduced from 14.9 to 1.6%. Impact in individual sites ranged from 57 to 100% reductions in parasite prevalence between 2004 and 2016. Sleeping under a net and living in a house that had been sprayed in the past 12 months were independently protective against infection (OR = 0.69 [95%CI 0.61–0.80] and OR = 0.87 [95% CI 0.78–0.97], respectively), whilst recent travel to the mainland increased the odds of infection nearly fourfold (OR = 3.94 [95%CI 2.79–5.56]).

**Conclusion:**

Island-wide interventions have resulted in a substantial reduction in malaria transmission on Bioko Island. This unique time series of 13 consecutive annual malaria indicator surveys clearly demonstrates the long-term effectiveness of the sustained use of two vector control interventions, indoor residual spraying and LLINs, and the value of comprehensive and sustained surveillance. Despite considerable success in reducing the burden on the island, malaria is still endemic, with populations in some areas remaining at high risk of infection.

**Electronic supplementary material:**

The online version of this article (10.1186/s12936-018-2213-9) contains supplementary material, which is available to authorized users.

## Background

Despite a large scale-up in access to long-lasting insecticide-treated nets (LLIN) and indoor residual spraying (IRS), malaria remains a major public health burden across much of sub-Saharan Africa. As intervention coverage increases across the continent, monitoring and evaluation of control programmes become paramount, although reliable indicators are often lacking and can be contradictory [1]. Several recent studies have used repeated community surveys to analyse long-term trends of malaria transmission in areas of declining exposure to malaria parasites [2–4]. Community surveys have advantages over passively collected data in that they are able to estimate the true prevalence within a population, regardless of symptoms and health-seeking behaviour. Accurately tracking changes in malaria transmission following implementation of malaria control can help to ensure that interventions are efficiently targeted.

Islands are considered ideal settings for effective malaria control due to the limited population migration and well-defined geographical borders. Several island nations have achieved substantial reductions in malaria transmission attributed to successful interventions [5–8], with Sri Lanka recently being certified malaria-free [9]. Bioko Island, Equatorial Guinea, with historically high malaria transmission [10], has been subject to extensive interventions including intensive vector control, improved case management, intermittent preventative treatments (IPT) and behavioural change interventions since 2004 through the Bioko Island Malaria Control Project (BIMCP) [11]. The BIMCP is a public–private malaria control project funded by Marathon Oil, the government of Equatorial Guinea and other partners. It has been implemented by Medical Care Development International (MCDI), along with researchers and key stakeholders. Early evaluations indicated that malaria transmission had dropped considerably, resulting in a 60% reduction in child mortality following the first 4 years of interventions [12], and a corresponding 50% drop in parasite prevalence in children aged between 1 and 14 years [13]. However, despite its island status, a study in 2013 demonstrated that travel to the mainland of Equatorial Guinea increased the risk of infection in travellers themselves, and in non-travellers living in neighbourhoods with high proportions of travellers [14], highlighting the impact of population movement on malaria transmission levels [15–18].

Malaria indicator surveys (MIS) take place annually within sentinel sites across the island as part of an enhanced monitoring and evaluation programme to assess the progress of the BIMCP towards its ultimate goal of malaria elimination. Transmission trends over more than a decade of vector control were examined using the time series of 13 consecutive cross-sectional assessments of parasitaemia and anaemia prevalence.

## Methods

### Setting and interventions

Bioko, the largest island of Equatorial Guinea (approximately 2000 km^2^), is 32 km from the coast of Cameroon. Its population, of approximately 250,000 (of which approximately 90% live in Malabo, the capital city,) are at risk of malaria year-round. Before the launch of the BIMCP in 2004, entomological inoculation rates (EIR) on the island were in excess of 750 infectious bites per person per year [10].

Island-wide Indoor Residual Spraying (IRS) was the principal intervention implemented by the BIMCP on a biannual basis beginning in 2004. The insecticide deltamethrin was used in the first year, followed by bendiocarb from 2005 to 2012, then deltamethrin again from 2013 to 2014 with a switch back to bendiocarb in 2015 and 2016. Since the switch back to bendiocarb in 2015, IRS has been targeted at particular communities rather than the whole island, as a strategy for enhancing the cost-effectiveness of vector control, taking into account variable community acceptance of the intervention after a decade of persistent spraying. Targeting was based on a risk score composed of community-level parasite prevalence, quality of housing (a measure of susceptibility to vector infiltration), risk of parasite importation as expressed by prevalence of reported travel to the mainland within the preceding 8 weeks, and previous IRS coverage (as a proxy for acceptability in the community).

A first mass distribution of long-lasting insecticide-treated nets (LLIN) was conducted on Bioko in late 2007 and early 2008, which achieved initial reported coverage of an average of three nets per household [19]. A second mass distribution of LLIN took place in late 2014 and early 2015, reportedly reaching 87% of households (unpublished data, BIMCP).

### Monitoring and evaluation

MIS have been conducted annually in August/September on Bioko since 2004. This study utilizes data from 13 surveys from 2004 through to 2016. The survey protocol has stayed broadly similar throughout this time period with the exception of the age group targeted for sampling. Between 2004 and 2007, only children aged between 1 and 14 years were sampled, with the addition of head of households between 2008 and 2012, and the inclusion of everyone present in the household from 2013 onwards.

**Fig. 1:**
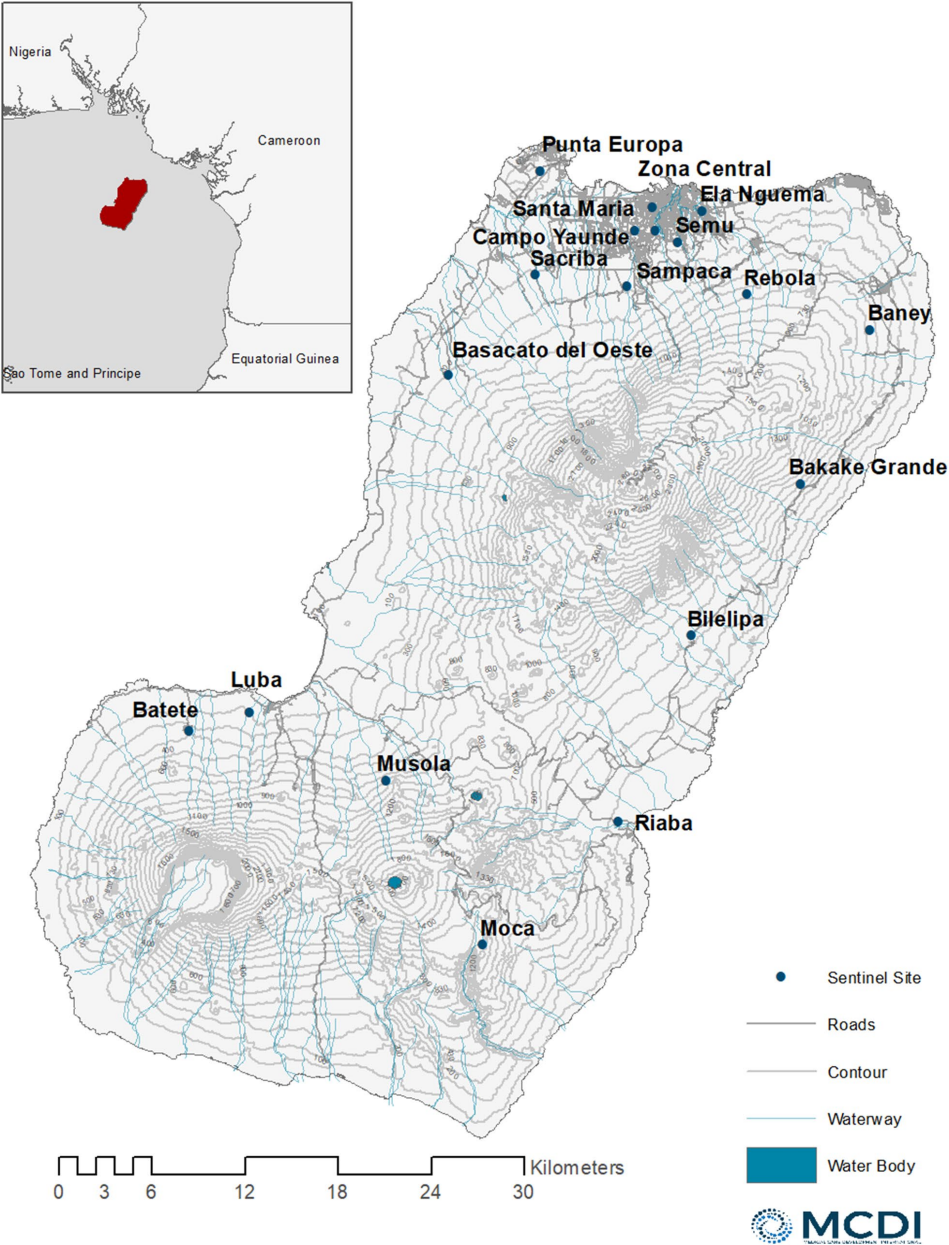
Map showing Bioko Island and the location of the 18 sentinel sites

Seventeen sentinel sites were monitored in 2004, increasing to 18 sites (with the addition of Punta Europa) from 2005 onwards (Fig. 1). The study was originally powered to detect a decline of 30% in each sentinel site between years, assuming a design effect of 2.5. In the two most recent surveys (2015 and 2016), the sampling frame switched to include all 284 communities on the island, with over-sampling in the smaller of the sentinel sites to ensure historical comparison over time. The analysis presented here is restricted to households sampled within sentinel sites to allow for assessment of longitudinal trends. Households within sentinel sites were randomly sampled using systematic sampling from a household listing. Heads of households were asked whether their house had received IRS within the previous 12 months and whether individuals in their household used a net the night prior to the survey. From 2013 onwards, data on travel to mainland Equatorial Guinea within the previous 8 weeks was collected for each household member.

Haemoglobin level was measured using Haemocues (HemoCue AB): mild anaemia was classified as a haemoglobin level less than 12 g/dl, 11.5 g/dl and 11 g/dl for individuals over 12 years, between 5 and 12 years and under 5 years, respectively. Moderate to severe anaemia was classified as a haemoglobin level below 8 g/dl for all age groups. Participants were tested for the presence of parasitaemia using a Pf/pan rapid diagnostic test (RDT) (ICT diagnostics) from 2004 to 2011 and Carestart^®^ RDT (Access Bio) in the later surveys. Participants testing positive were referred to a survey nurse for treatment, according to national treatment guidelines (artestunate/amodiaquine), whilst participants with low haemoglobin levels were advised to seek care.

### Statistical analyses

Due to the restricted age groups in the early surveys, only results from children aged 1–14 years are included in figures and analyses. Prevalence of infection by RDT and 95% confidence intervals were calculated for each year. The association between parasite prevalence and prevalence of moderate/severe anaemia (< 8 g/dl) in children aged 1–5 years was assessed using regression models. Differences in proportions were assessed using Chi^2^ tests. A logistic regression model, allowing for the survey design, was performed to estimate the mean effect of time (year; linear and categorical) on infection prevalence.

Median altitude of each sentinel site was calculated based on household global positioning system (GPS) information and sites were classified as high altitude if they were above the median altitude for all sites (108 m). Site-level spray coverage (based on reported spraying within sampled households) was estimated to investigate benefits of high IRS coverage at the community level. Households were classified as having universal coverage of nets if they reported having at least one net for every two people in the household. To investigate the impact of original malaria endemicity, the median baseline site prevalence (45%) was used to stratify sites into high or moderate prevalence (< 45%).

Information about intervention coverage (net use and IRS within the last 12 months) was collected from 2006 onwards. Univariate logistic regression was used to look at the differential effects of intervention coverage on infection, comparing (1) living in a sprayed household and not using a net (2) reporting sleeping under a net and not living in a sprayed house (3) living in a sprayed house and sleeping under a net, to a baseline of no interventions. Variables that showed a crude association with RDT positivity were included in the model in a stepwise manner, including age group, original site prevalence, and site-level spray coverage. To investigate whether the switch in IRS strategy had an effect on risk factors, models were examined with and without the surveys from 2015 to 2016. A separate model including recent travel to the mainland was used for data from surveys from 2013 to 2016. For consistency, this model also only included children aged 1 to 14 years.

Sampling probabilities of children aged between 1 and 14 years in each sentinel site were computed using data from a country-wide 2015 census. Responses were weighted by the inverse of sampling probabilities to compensate for the different numbers of children tested in each survey. All models controlled for year to account for temporal variations. Standard errors were adjusted to account for correlation of responses, with sentinel site specified as the primary sampling unit (PSU) when calculating an island-wide prevalence and household when calculating sentinel site level prevalence.

## Results

In the thirteen surveys between 2004 and 2016, 35,146 household visits were conducted, ranging from 1043 to 4721 households per year (Table 1). The median household size was 6, with an average of 2 children (aged between 1 and 14 years) tested per household.

**Table 1:** Demographics and intervention access and use in the study population

Year	No. of households	No. of people tested	Females tested (%)	Age (median and range)	Children aged 1–14 years (% of all data collected)	% of households that reported spraying within the previous 12 months [95% CIs]	% of households with universal net coverage (at least 1 net per 2 people) [95% CIs]	% of participants aged 1–14 reporting sleeping under a net the previous night [95% CIs]	% of participants aged 1–14 living in a household with universal coverage reporting sleeping under a net the previous night [95% CIs]
2004	1043	2541	nd	6 (1–15)	2525 (99.5)	0.0	nd	nd	nd
2005	1387	3809	nd	5 (0–14)	3435 (90.2)	80.4 [72.7, 86.3]	nd	nd	nd
2006	2312	5395	2755 (51.3)	6 (1–14)	5356 (99.3)	77.3 [72.9, 81.2]	16.5 [11.2, 23.6]	31.8 [22.1, 43.3]	86.4 [79.1, 91.4]
2007	1937	4745	2416 (51.0%)	5 (0–14)	4472 (94.3)	60.2 [56.5, 63.8]	17.1 [13.6, 21.2]	33.5 [26.2, 41.8]	88.7 [84.4, 92.0]
2008	2994	7346	4146 (56.6)	10 (0–102)	4403 (59.9)	76.5 [73.1, 79.7]	57.0 [53.5, 60.5]	81.3 [75.4, 86.1]	89.3 [85.0, 92.5]
2009	2245	7848	4398 (58.4)	8 (0–88)	5774 (73.6)	76.9 [73.2, 80.3]	20.5 [17.2, 24.1]	39.7 [32.6, 47.2]	89.8 [84.3, 93.6]
2010	2938	10,082	5778 (58.6)	8 (0–94)	7402 (73.4)	64.9 [60.8, 68.8]	18.2 [15.5, 21.3]	33.1 [27.3, 39.6]	85.6 [79.4, 90.2]
2011	2818	8511	4758 (58.9)	8 (0–91)	6155 (72.3)	67.9 [62.6, 72.7]	13.4 [11.2, 15.9]	24.6 [18.8, 31.6]	91.9 [88.1, 94.6]
2012	2791	9159	5302 (59.5)	8 (0–95)	6511 (71.1)	70.9 [66.9, 74.7]	16.5 [13.9, 19.4]	28.2 [24.6, 32.1]	86.7 [80.3, 91.3]
2013	4721	14,792	8330 (56.4)	14 (0–101)	7137 (48.3)	76.1 [72.9, 79.1]	22.4 [19.9, 25.1]	32.1 [26.8, 37.8]	83.3 [74.5, 89.6]
2014	4528	15,958	9004 (56.6)	13 (0–100)	8253 (51.7)	75.3 [70.5, 79.6]	20.6 [17.6, 23.9]	34.2 [28.5, 40.3]	85.7 [81.0, 89.4]
2015	2732	8456	4775 (56.6)	15 (0–97)	4013 (47.5)	55.9 [52.1, 59.7]	42. [38.8, 46.1]	54.2[47.3, 61.0]	79.9 [71.9, 86.1]
2016	2700	7921	4152 (52.4)	17 (0–96)	3411 (43.1)	61.5 [49.9, 72.0]	40.4 [36.0, 45.0]	47.4 [41.3, 53.6]	76.0 [67.9, 82.6]

*nd* data not collected

Reported spray coverage ranged from 56% (in 2015 when targeted focal IRS was introduced) to 80% (in 2005). Spray coverage varied substantially between sites. Sites in Malabo and the North West consistently did not achieve spray coverage over 80%-in part due to a large proportion of refusals from homeowners. A low proportion of houses reported universal net coverage, ranging from 13% (in 2011) up to 57% and 42%, following the LLIN campaigns in 2007/08 and 2014/15, respectively. In the subsequent year, following the first distribution, universal coverage had already substantially reduced (21%) whilst coverage remained similar in the year following the second distribution (40%). The percentage of children aged 1–14 years sleeping under a net the night before the survey was below 55% in all survey years except in 2008 when it increased to 81% (Table 1). By 2009, usage had already decreased to 40%. Utilization increased to 54% following the 2nd mass distribution in 2014/15, though it remained substantially lower than immediately after the 1st mass distribution in 2007/08. Utilization of nets was higher in houses that had universal coverage, with usage varying between 76% and 92% over the study period (Table 1). Within these households, priority for sleeping under nets appeared to be given to the youngest children, with 85% of under 5 s sleeping under a net, compared to 82% and 78% of 5–10 and 10–14 year olds, respectively (p < 0.001). In houses without universal coverage, priority was also given to children aged between 1 and 5 years, with 30% reporting sleeping under a net, compared to only 23% and 20% of children aged 5–10 and 10–14, respectively (p < 0.001). In participants over the age of 14 years, 27% reported using a net in households without universal coverage, compared to 78% in households with universal coverage.

### Prevalence of parasitaemia

Island-wide *P. falciparum* prevalence, detected by RDT, decreased by over 75% between 2004 and 2016, from 43.3% (95%CI 37.5–49.3%) to 10.5% (8.8–12.5) (p < 0.001) (Table 2). On average, there was an 11% decrease in the odds of infection per year (OR 0.89 (0.87–0.90), p < 0.001). However, the decrease was not uniform, with similarly steep reductions seen in the first year following the implementation of interventions (2005 OR 0.55 (0.44–0.70) (compared to 2004) and in 2014 (OR 0.54 (0.43–0.68) compared to 2013) (Table 2). Several years saw no change in prevalence from the previous year (2007, 2009, 2010) whilst in 2013 there was a notable increase in prevalence.

**Table 2:** Weighted prevalence of moderate/severe anaemia (< 8 g/dl) in children aged 1–5 years and weighted parasitaemia and risk of infection compared to baseline by year, in children aged 1–14 years

Year of survey	No of children aged 1–5 tested for anaemia	% of children aged 1–5 years with severe anaemia [%, 95% CIs]	No. of children (aged 1–14 years) tested for parasitaemia	Parasite prevalence (%, 95% CI)	Crude odds ratio [95% CI]	Crude odds ratio (using previous year as baseline)
2004	1129	14.9 [12.3, 18.0]	2525	43.3 [37.5, 49.3]	1	–
2005	1648	9.4 [7.0, 12.6]	3435	29.7 [23.4, 36.9]	0.55 [0.44,0.70]	0.55 [0.44,0.70]
2006	2297	6.8 [5.4, 8.6]	5356	26.8 [22.9, 31.2]	0.48 [0.34,0.67]	0.87 [0.63-1.19]
2007	2130	7.3 [5.5, 9.8]	4472	29.2 [24.9, 33.8]	0.54 [0.44,0.66]	1.12 [0.84-1.50]
2008	2166	1.9 [1.6, 2.3]	4403	19.0 [16.1, 22.4]	0.31 [0.22,0.44]	0.57 [0.41-0.79]
2009	2697	2.7[1.8, 3.9]	5774	21.3 [14.8, 29.8]	0.36 [0.24, 0.52]	1.15 [0.73–1.81]
2010	3403	2.8 [2.2, 3.6]	7402	24.4 [18.5, 31.5]	0.42 [0.32, 0.57]	1.19 [0.96–1.48]
2011	2931	2.9 [2.2, 3.9]	6155	18.9 [13.9, 25.2]	0.31 [0.25, 0.38]	0.72 [0.54–0.97]
2012	3003	2.7 [2.2, 3.3]	6511	12.7 [10.1, 15.9]	0.19 [0.16, 0.23]	0.62 [0.51–77]
2013	3379	3.3 [2.6, 4.3]	7137	27.5 [20.8, 35.5]	0.50 [0.39, 0.64]	2.61 [2.18–3.13]
2014	3543	2.6 [2.1, 3.4]	8253	17.0 [13.8, 20.8]	0.27 [0.21, 0.34]	0.54 [0.43–0.68]
2015	1866	1.5 [1.0, 2.1]	4013	13.8 [10.7, 17.7]	0.21 [0.15, 0.28]	0.78 [0.61–1.00]
2016	1505	1.6 [0.9, 2.6]	3411	10.5 [8.8, 12.5]	0.15 [0.12, 0.20]	0.73 [0.58–0.92]

*Plasmodium falciparum* prevalence decreased across all age groups over the study period (see Additional file 1). Children aged 5–14 years were consistently most likely to be infected. In 2004, the burden fell primarily on 5 to 10 year olds, but by 2016, this had shifted to 10–14 year olds. From 2013 onwards, participants aged 15–25 years were also at higher risk than under 5s (ORs ranging from 1.3 to 1.7).

### Prevalence of anaemia

Prevalence of moderate/severe anaemia (haemoglobin < 8 g/dl) in children aged 1 to 5 years dropped by nearly 90% between 2004 and 2016 (15–2%) (Table 2). All sites apart from Rebola achieved at least a 75% reduction in the prevalence of anaemia, with several sites recording no cases of severe anaemia in under-fives tested in 2016. In Rebola, anaemia increased from 2.5% in 2004 to 4.7% in 2016.

**Fig. 2:**
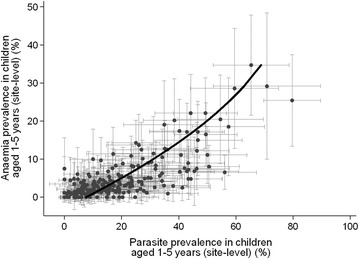
Correlation between site level prevalence of severe anaemia (< 8 g/dl) and parasite prevalence in children aged between 1 and 5 years, with 95% confidence intervals. The data is overlaid with a quadratic fit, indicating that parasite prevalence increases by 2.5% (1.8–3.3%) for every 1% increase in anaemia, with the slope decreasing by 0.04% for every 1% increase

Approximately 12% of children aged 1–5 who were parasite positive had moderate/severe malaria, compared to 2% who were not parasite positive (p < 0.001). Site-level anaemia prevalence was strongly correlated with parasite prevalence (Fig. 2).

### Heterogeneity of intervention effects

**Fig. 3:**
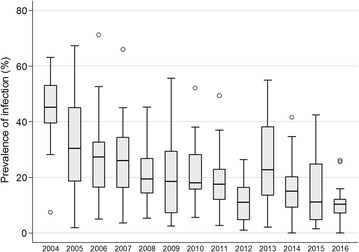
Box and whisker plot highlighting site-level parasite prevalence in children aged 1–14 years by year. The mid-line in the box represents the median prevalence across all sites

**Fig. 4:**
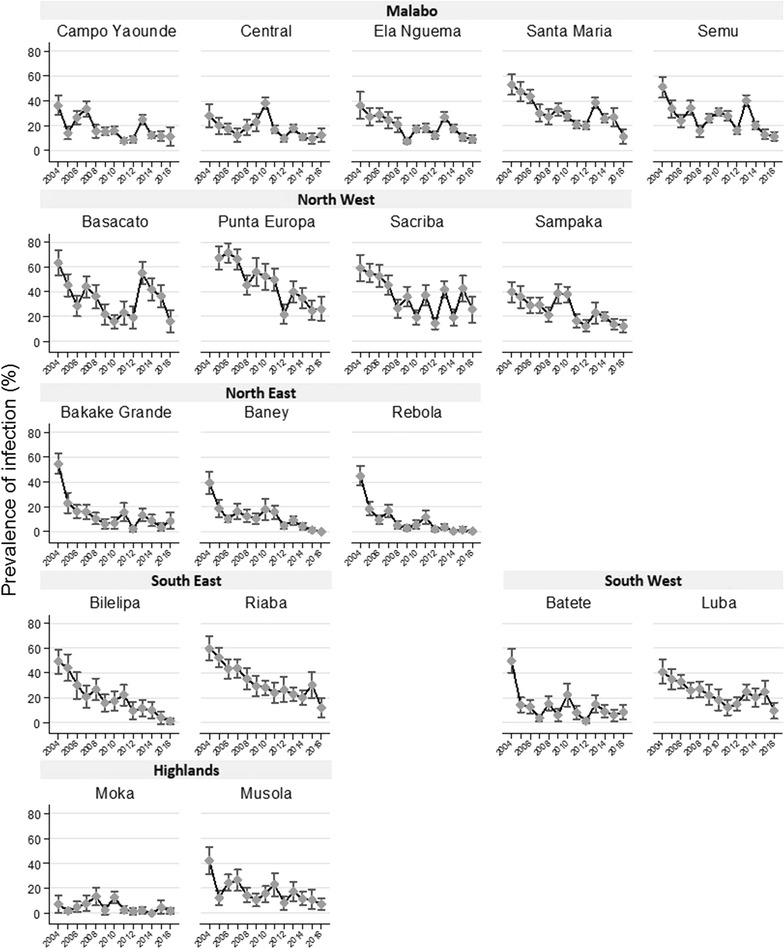
Parasite prevalence in children aged 1–14 years for 18 sentinel sites from 2004 to 2016. Sites are ordered by their location on the island

**Fig. 5:**
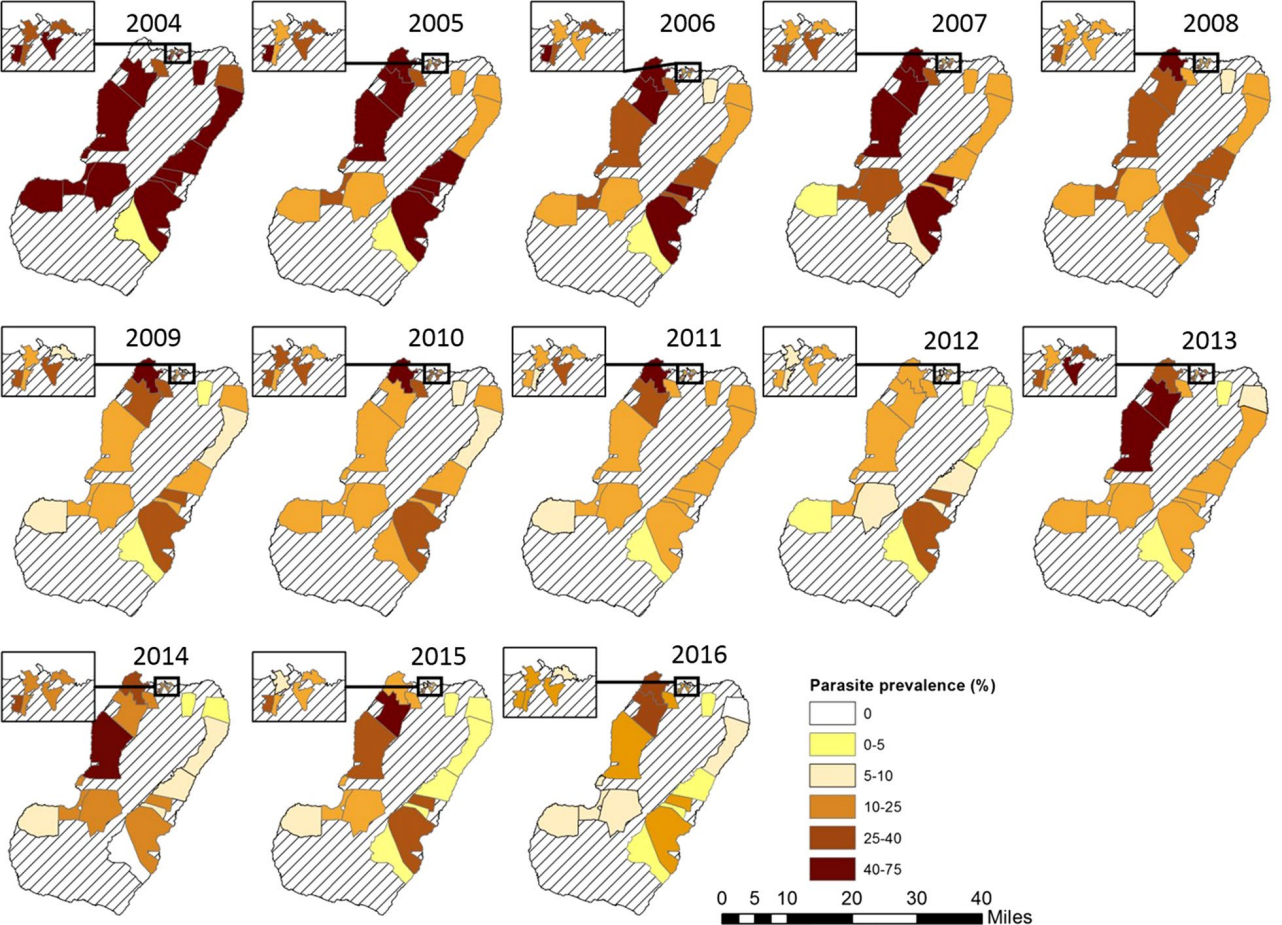
Maps showing parasite prevalence in children aged 1–14 years by sentinel site between 2004 and 2016

Whilst prevalence of infection reduced over time, there were differences between sites (Fig. 3) and heterogeneity in the reduction of transmission over the 13 surveys (Figs. 4, 5). The biggest reductions between 2004 and 2016 were seen in Baney, where parasite prevalence was 0% in 2016 (compared to 40% in 2004), Rebola (a 99% decrease since 2004) and Bilelipa (a 98% decrease). The smallest reductions were seen in Sacriba and Central (57% decrease since 2004) and Punta Europa (61% decrease (since 2005)). However, all three sites had recorded lower prevalence in 2012, which subsequently rebounded. For instance, in Sacriba in 2012, prevalence had decreased by 76% since 2004, but these gains were lost in 2013 when prevalence rebounded from 14 to 37% (Figs. 3, 4, 5). Sites in rural areas recorded more substantial reductions in prevalence between 2004 and 2016 than sites in urban areas (79.0% vs. 74.1%).

The 3 sites which had achieved highest impact were compared to those that had achieved the least impact to establish whether there were key differences. Participants who lived in the sites with the highest impact were less likely to have travelled (2.3% vs. 9.2%, p = 0.01) and were more likely to live in sprayed households (79.7% vs. 67.5%, p = 0.037). Net use and access was similar in all six sites. Sites with highest impact had median altitudes over 200 m whilst lowest impact sites all had median altitudes lower than 55 m. Two out of three sites with the highest impact were classified as moderate prevalence pre-intervention (Baney and Rebola), whilst only one of the least impact was classed as moderate prevalence (Central).

In 2016, 9 of the 18 sentinel sites had prevalences below 10%: this included all sites in the North East, South West and Highland regions of the island. No sites in the North West region (Basacato, Punta Europa, Sacriba and Sampaka) had parasite prevalences under 10% (Fig. 5).

### Risk factors for parasite positivity

There was no evidence for risk factors varying substantially between years. There was not strong evidence that living in a site with high spray coverage (> 80%) or in a site with higher altitude was associated with lower infection prevalence (Table 3). The association between spray coverage and infection prevalence remained even when 2015 and 2016 (when spraying was targeted) were removed from the analysis. The final model included age group, pre-intervention site prevalence, and vector control usage. Under 5s, living a site with lower initial prevalence, and net use in a recently sprayed house were all associated with lower odds of infection (Table 3). Sleeping under a net reduced the odds of infection by a quarter [OR 0.75 (0.63–0.88)] whilst sleeping in a recently sprayed house reduced the odds by 14% [OR 0.86 (0.77–0.96)], compared to no interventions. There was an additive effect of the two interventions [OR 0.58 (0.53–63)] and no evidence of interaction between the two interventions (p = 0.178).

**Table 3:** Risk factors for *P. falciparum* infection in children aged 1 to 14 years from 13 years of survey data

Risk factors		% RDT positive (N)	Odds ratios (OR)^a^	95% CI	p value	Adjusted Odds Ratios (aOR)^b^	95% CI	p value
Intervention use								
No intervention		24.4% (13,871)	1			1		p < 0.001
Sleeping under a net		17.6% (4826)	0.69	(0.61–0.80)	p < 0.001	0.75	(0.63–0.88)	
Sleeping in a sprayed house		22.1% (26,351)	0.87	(0.78–0.97)		0.86	(0.77–0.96)	
Slept under a net and sleeping in a sprayed house		15.7% (14,867)	0.57	(0.52–0.63)		0.58	(0.53–0.63)	
Age group								
1–5 years		20.8% (32,077)	1		p < 0.001	1		p < 0.001
5–10 years		28.9% (22,952)	1.59	(1.43–1.78)		1.70	1.60–1.80	
10–14 years		29.9% (13,818)	1.67	(1.53–1.84)		1.95	1.78–2.11	
Site location								
Living in an urban site		21.3% (35,141)	1					
Living in a rural site		21.6% (33,706)	1.0	(0.65–1.54)	0.962			
Spray coverage								
Living in a site with spray coverage < 80%		26.4% (50,754)	1					
Living in a site with spray coverage ≥ 80%		19.9% (18,093)	0.74	(0.46–1.2)	p = 0.214			
Site altitude								
Living in a site with median altitude < 108 m		23.9% (45,407)	1					
Living in a site with median altitude ≤ 108 m		16.7% (23,482)	0.79	(0.47–1.3)	0.345			
Previous transmission level								
Living in a site with low prevalence in 2004 (< 45%)		21.4% (39,016)	1			1		
Living in a site with high prevalence in 2004 (> 45%)		31.3% (29,831)	1.72	(1.31–2.26)	p < 0.001	1.66	1.22–2.26	0.003

^a^Adjusted for survey year

^b^Adjusted for age group, original site prevalence and year

The influence of recent travel to the mainland was examined for surveys where data was collected (2013–2016). The proportion of participants reporting travel was relatively small, ranging from 6% in 2013 and 2014, up to 10% in 2016, but with wide variation between sites. Travellers were more likely to be women and to be aged between 15 and 50 years. The proportion of people travelling was highest in sites in Malabo, increasing from 9% in 2013 to 14% in 2016. Infection was higher in people reporting recent travel than those who had not travelled (30.1% vs. 12%, p < 0.001). Before adjusting for other risk factors, travel to the mainland increased the odds of infection in children aged 1–14 nearly 4-fold (OR 3.6 (2.6–5.1, p < 0.001)). When incorporated with the other risk factors in the final model, travel to the mainland remained strongly associated with infection (OR 3.4 (2.5–4.6, p < 0.001)).

## Discussion

Monitoring and evaluation play a key role in malaria control campaigns and are essential for assessing the impact of interventions and guiding programmes in adapting operational approaches. The World Health Organization (WHO) Global Technical Strategy [20] emphasizes surveillance as a core intervention for reducing malaria burden and achieving elimination. On Bioko, comprehensive surveillance through annual MIS have allowed for detailed examination of the impact of interventions and control strategies.

The BIMCP has been an overall success, achieving a reduction in parasitaemia of over 75% over 13 years. In the early years of implementation, there was heterogeneity of impact between sites. Differential impact of universal interventions have been documented in other studies [4, 21] and has been attributed in part to uneven distribution or uptake of interventions [11, 22], which was seen on Bioko with varying coverage of IRS and use of LLINs. There was evidence that living in a site that had initial high prevalence resulted in a higher risk of infection overall, although, this was not universally true, with Bilelipa (with a pre-intervention prevalence of 49%) recording a 97% reduction in prevalence by 2016, whilst Central (pre-intervention prevalence of 28%) achieved a more modest 57% reduction. More notable was the difference in reduction by region with the North Eastern sites (Bakake Grande, Baney, Rebola) showing an average decrease of 94% compared to an average decrease of 65% in the North West (Basacato, Punta Europa, Sacriba, Sampaka). In Punta Europa, a site which consistently showed higher prevalence, this was attributed initially to low IRS coverage with infection prevalence decreasing once IRS coverage increased.

The main preventative intervention employed on Bioko is IRS. Whilst IRS can protect the sprayed household through a repellent effect (depending on the insecticide used), its main effect is through killing action resulting in community-wide protection when a sufficiently high spray coverage is reached [23]. The WHO recommends that > 80% of structures need to be sprayed for IRS to have a community effect [24]. In this study, living in a site with spray coverage over 80% resulted in lower risk of infection but the association was not significant. A previous study collating data from Equatorial Guinea and Malawi demonstrated that higher spray coverage resulted in decreased infection prevalence [19].

In addition to IRS, two mass LLIN distributions took place; however, MIS which took place within 6 months of the distributions suggest that if high coverage was originally achieved, it had quickly reduced. This may be because some of the nets that were distributed were subsequently taken to the mainland where little malaria control is in place and malaria transmission is substantially higher. Studies are ongoing to establish why attrition of nets was so high. In households with universal coverage, the use of nets was high, indicating that the barrier to higher LLIN utilization is the lack of access to nets, not unwillingness to use them.

Net use was shown to be protective against infection, with children reporting sleeping under a net the previous night (and not living in a sprayed house) having 30% lower odds of being infected. A treated net provides a physical barrier against biting mosquitoes and protection through reduction of the mosquito population by insecticidal action. Several sites saw steep falls in parasite prevalence between 2007 and 2008, which may have been due to increased net use. The proportion of people with access to nets post-2008 reduced to approximately 20%, but despite this, there was no sign of a rebound in malaria prevalence (or severe anaemia), suggesting other factors were also contributing to maintenance of the new lower equilibrium. The highest infection prevalence was consistently detected in children aged between 5 and 14 years old, who were also least likely to be using a net.

IRS and LLIN are often used in combination rather than alone. As with previous studies in Equatorial Guinea [25, 26], and elsewhere [27, 28], this study demonstrated an additive effect of the combination of both interventions. The effect of the combination of the two interventions is likely to depend on intervention coverage, initial transmission levels, the insecticides used, and on the local vector population [29–31].

In recent years, population movement to the mainland of the country increased, perhaps due to more transport options, or an overall increase in wealth. Travel to the mainland, where malaria is higher [26], has been shown to be a risk factor for infection on Bioko previously [14]. More people reported travel in the three sites demonstrating the least impact, compared to the three with the highest impact, further highlighting the importance of this risk factor. Infection importation remains a persistent and significant challenge for malaria control on Bioko and a potential barrier to further reduction of transmission on the island. Despite being an island, population travel to and from higher risk areas makes Bioko vulnerable to importation of infection. Border screening and treatment, targeted ‘Malaria Awareness’ campaigns or chemoprophylaxis may help to alleviate this issue. Expansion of control activities to include the mainland area of Equatorial Guinea are likely to have an additional impact.

Whilst interventions have reduced the risk of infection, substantial variation in transmission over the study period remains unexplained. A factor which may have resulted in reduced transmission over time is an increase in the quality of housing, which has been shown in Bioko [32], and elsewhere, to be associated with lower malaria incidence [33, 34]. Bioko has seen improvements in socio-economic status during the project timeline which has probably also contributed to a reduction in overall transmission. In addition, access to free treatment with artemisinin-based combination therapy was introduced in 2005 and provision of drugs through private vendors has also increased over the study period. A reason for the differential impact could be due to population behaviour, such as population movement between higher and lower transmission areas or increasing outdoor behaviour in the evenings due to the provision of electricity and lighting resulting in heightened exposure to outdoor biting. Data collected several years ago on Bioko suggested that outdoor biting was not associated with malaria infection at the time, mainly because peak biting occurs at a time when most people are indoors [35]. However, as human behaviour changes, outdoor biting may become a significant risk for infection.

Although there has been a decrease in prevalence in most years of the project, 2013 stood out as an anomaly, when prevalence increased in most sites, following a particularly low prevalence recorded in 2012. This may have been influenced by the choice of insecticide used that year. In 2004, the insecticide used for spraying was switched from deltamethrin to bendiocarb following the detection of the *kdr* mutation in the *Anopheles gambiae* population [36]. Subsequent analysis showed that despite the presence of *kdr*, the 2004 pyrethroid spray round nevertheless resulted in a sharp reduction in sporozoite infectivity in the main vector after spraying, and there was no apparent association between *kdr* and phenotypic resistance. In 2013 deltamethrin spraying was re-introduced as part of a programme of insecticide resistance management through rotation [37]. Following the change back, a sharp increase in parasite prevalence was recorded in the subsequent household survey of 2013, despite no obvious decrease in intervention coverage, prompting fears of resistance of local vectors to deltamethrin [38]. The presence of resistance markers is currently being monitored across the island.

Despite the heterogeneous impact of interventions in Bioko, transmission has declined substantially overall. Understanding the factors that have contributed to differential impact will help the BIMCP to prioritize strategies and to continue the downward trajectory in parasite rate. Indeed, several areas, which have now reached pre-elimination levels, are now implementing further strategies, such as reactive case detection, to attempt elimination of local transmission. This study has shown that vector control with IRS and LLINs remained the most important protective intervention on Bioko, whilst travel to the mainland constituted a substantial risk for malaria infection. The low proportion of houses with universal coverage of LLINs, and data suggesting that the age group most likely to be infected are least likely to be using a net (10–14 yr olds) is concerning. Strategies to ensure a higher proportion of the population have access to nets have been implemented, but the lack of LLINs on the mainland and the high rate of associated leakage of nets, may have undermined the ability to maintain high coverage levels. Controlling parasite importation from the mainland, and maintaining universal access to the core prevention, namely LLIN use, represent two clear challenges to further reducing malaria transmission on Bioko.

Whilst the gains over the past 13 years have been impressive, the slow, but steady progress highlights the importance of sustaining malaria control interventions on a long-haul basis. Long-term commitment and surveillance are necessary to chart the downward trend which may not necessarily advance in an uninterrupted linear manner.

## Additional file

**Additional file 1: Figure S1.** Parasite prevalence and 95% confidence intervals by year of age between 2004 and 2016.
