# Supplementary figures and images for: Trends in parasite prevalence following 13 years of malaria interventions on Bioko island, Equatorial Guinea: 2004–2016
Source: Malar J. 2018 Feb 5;17:62. doi: 10.1186/s12936-018-2213-9 (PMC5799938; doi:10.1186/s12936-018-2213-9)

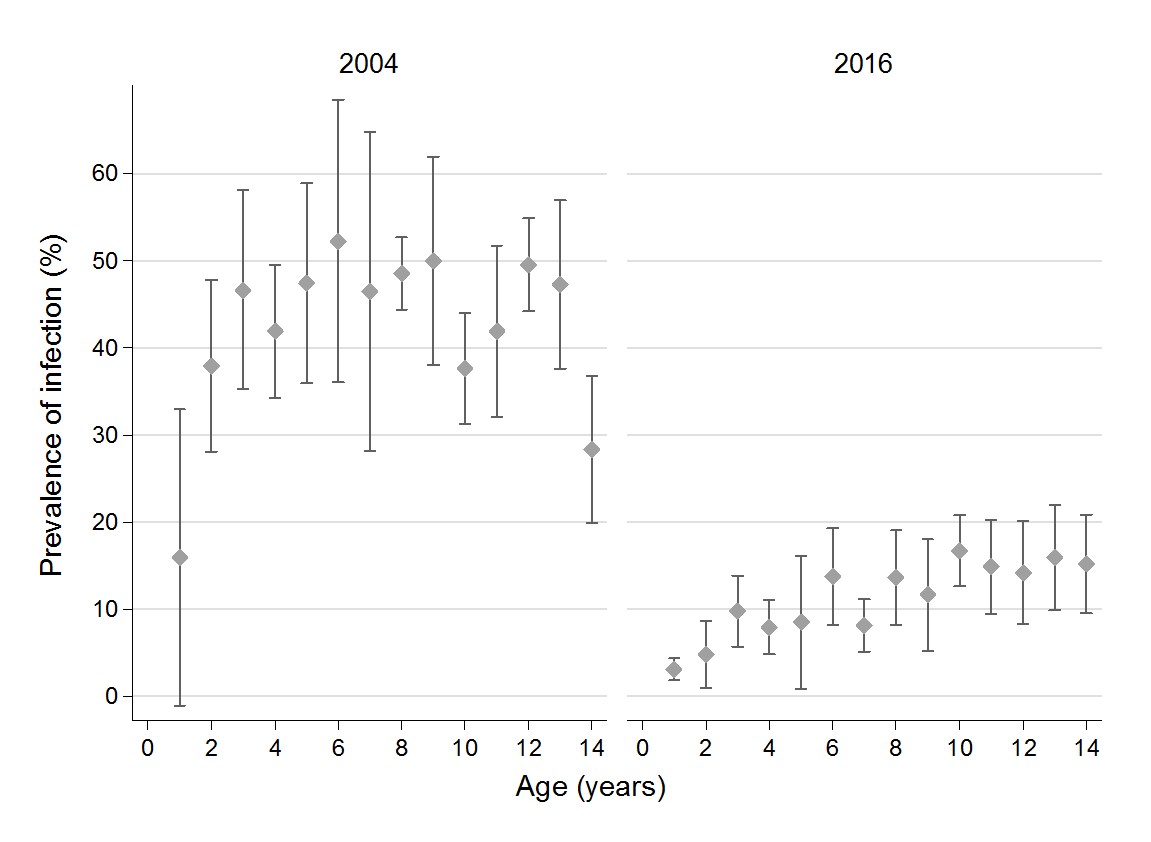

Supplement: Supplementary file 1 — Additional file 1: Figure S1. Parasite prevalence and 95% confidence intervals by year of age between 2004 and 2016. [file 12936_2018_2213_MOESM1_ESM.jpg]
